# A novel gene expression system strongly enhances the anticancer effects of a REIC/Dkk-3-encoding adenoviral vector

**DOI:** 10.3892/or.2013.2958

**Published:** 2013-12-31

**Authors:** MASAMI WATANABE, MASAKIYO SAKAGUCHI, RIE KINOSHITA, HARUKI KAKU, YUICHI ARIYOSHI, HIDEO UEKI, RYUTA TANIMOTO, SHIN EBARA, KAZUHIKO OCHIAI, JUNICHIRO FUTAMI, SHUN-AI LI, PENG HUANG, YASUTOMO NASU, NAM-HO HUH, HIROMI KUMON

**Affiliations:** 1 Center for Innovative Clinical Medicine, Okayama University Hospital, Okayama, Japan; 2 Department of Urology, Okayama University, Okayama, Japan; 3 Department of Cell Biology, Okayama University, Okayama, Japan; 4 Department of Medical and Bioengineering Science, Okayama University, Okayama, Japan; 5 Department of Veterinary Nursing and Technology, Nippon Veterinary and Life Science University, Musashino, Tokyo, Japan

**Keywords:** gene expression, human telomerase reverse transcriptase enhancer, Simian virus 40 enhancer, cytomegalovirus enhancer, adenovirus

## Abstract

Gene expression systems with various promoters, including the cytomegalovirus (CMV) promoter, have been developed to increase the gene expression in a variety of normal and cancer cells. In particular, in the clinical trials of cancer gene therapy, a more efficient and robust gene expression system is required to achieve sufficient therapeutic outcomes. By inserting the triple translational enhancer sequences of human telomerase reverse transcriptase (hTERT), Simian virus 40 (SV40) and CMV downstream of the sequence of the BGH polyA, we were able to develop a novel gene expression system that significantly enhances the expression of the genes of interest. We termed this novel gene expression cassette the super gene expression (SGE) system, and herein verify the utility of the SGE cassette for a replication-deficient adenoviral vector. We newly developed an adenoviral vector expressing the tumor suppressor, reduced expression in immortalized cells (REIC)/Dickkopf-3 (Dkk-3), based on the CMV promoter-driven SGE system (Ad-SGE-REIC) and compared the therapeutic utility of Ad-SGE-REIC with that of the conventional adenoviral vectors (Ad-CMV-REIC or Ad-CAG-REIC). The results demonstrated that the CMV promoter-SGE system allows for more potent gene expression, and that the Ad-SGE-REIC is superior to conventional adenoviral systems in terms of the REIC protein expression and therapeutic effects. Since the SGE cassette can be applied for the expression of various therapeutic genes using various vector systems, we believe that this novel system will become an innovative tool in the field of gene expression and gene therapy.

## Introduction

Gene therapy has been utilized in many clinical trials for human cancer and has exhibited innovative and attractive therapeutic potential. Adenovirus-mediated gene delivery continues to be preferred for the treatment of cancer, as the vectors have a high transduction efficacy for the therapeutic gene, and due to the safety of the procedure when it is used for direct local injection (1,2). A gene expression promoter, such as the cytomegalovirus (CMV), Rous sarcoma virus (RSV) and CMV early enhancer/chicken β-actin (CAG)promoters, has been used to increase the gene expression in a variety of normal and cancer cells, achieving favorable transduction efficiency (3–6). However, in some patients, the clinical cancer gene therapy using even these strong promoters has shown modest therapeutic effects (1). Some of these unfavorable results may be due to differences in the accomplishment of the robust and sufficient expression of the therapeutic gene for the treatment of the specific cancer type. Thus, particularly for clinical trials of cancer gene therapy, a more powerful gene expression system that can be used for a broad range of cancer types is urgently required.

A variety of enhancer sequences have thus far been reported and used for the modification and regulation of gene expression (7–11). In order to develop a novel system which enables more potent gene expression in comparison to the conventional systems, we have been modifying the construct sequence of plasmids by adding and exchanging the multiple known enhancer sequences. In a series of modifications with multiple enhancers in the CMV promoter-dependent gene expression cassette, we found that the insertion of the triple translational enhancer sequences of hTERT, SV40 and CMV downstream of the BGH polyA sequence led to the most potent gene expression. The human telomerase reverse transcriptase (hTERT) promoter/enhancer has been demonstrated to be available for cancer tissue-specific gene expression in a broad range of malignant cell types (12–14). The Simian virus 40 (SV40) and CMV promoter/enhancer have also been well characterized and have been used to improve the gene expression in mammalian cells (3,4,7–10). Since the novel gene expression system using the triple enhancers significantly enhances the expression of the gene(s) of interest in comparison to the conventional systems using the strong CMV promoter, we termed this novel gene expression cassette the super gene expression (SGE) system. We also call this cassette C-TSC (C, CMV promoter driving; TSC, enhancer unit composed of triple tandem enhancer sequences of hTERT, SV40 and CMV).

The reduced expression in immortalized cells (REIC) gene is a tumor suppressor that is identical to the Dickkopf-3 (Dkk-3) gene (15). The expression of REIC/Dkk-3 is significantly downregulated in a wide range of human cancer types, making REIC/Dkk-3 a promising cancer therapeutic gene (16–23). Expecting that transfection of REIC/Dkk-3 would provide therapeutic effects as a tumor suppressor gene, we previously developed an adenoviral vector expressing the human REIC/Dkk-3 gene (Ad-REIC) and demonstrated that the agent induced apoptosis in various cancer cell lines (16–18,23). In order to demonstrate the utility of the SGE system for cancer gene therapy, we developed an Ad-REIC vector with the SGE system (Ad-SGE-REIC). We then compared the Ad-SGE-REIC with the conventional Ad-REIC vectors, and herein report our evaluation and validation of the SGE system for the enhancement of gene expression and indicate its potential therapeutic utility.

## Materials and methods

### Cells and cell culture

Normal human hepatocytes (HC) were purchased from Applied Cell Biology Research Institute (Kirkland, WA, USA). Normal human fibroblasts (OUMS24) were established by the Department of Cell Biology at our university (15). All human cancer cell lines (PC3, prostate cancer; KPK1, renal cancer; 211H, malignant mesothelioma; HeLa, cervical cancer), the mouse renal cell carcinoma line (RENCA cells) and the HEK293 cell line were provided by the American Type Culture Collection (Rockville, MD, USA), and these cells were cultivated as previously described (16,21).

### Construction of plasmid vectors

**Figure 1:**
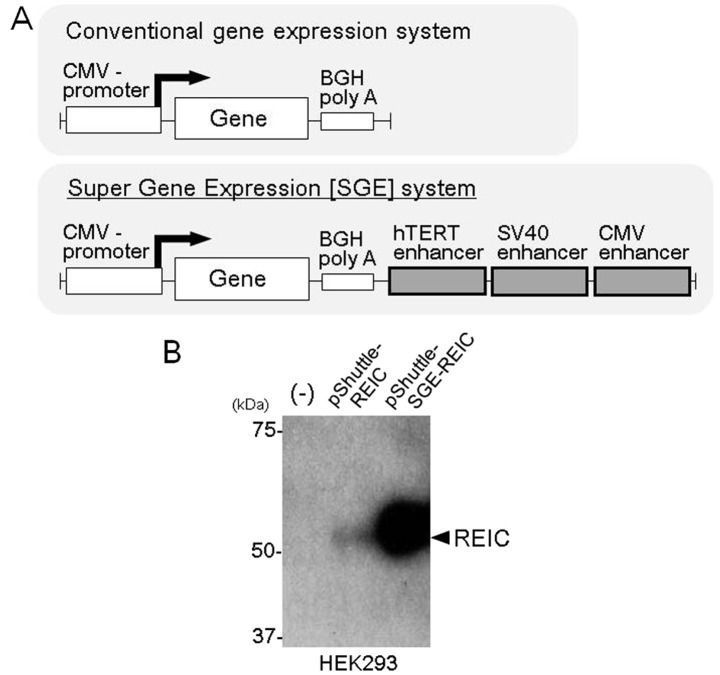
(A) A schematic diagram of the conventional gene expression system and the SGE system. In the SGE system, triple translational enhancer sequences (hTERT, SV40 and CMV) were inserted downstream of the BGH polyA sequence. (B) The pShuttle plasmid vector with the conventional system and SGE system encoding the REIC/Dkk-3 gene were termed pShuttle-REIC and pShuttle-SGE-REIC, respectively. The REIC/Dkk-3 expression levels after transfection with the pShuttle-REIC and pShuttle-SGE-REIC plasmids were compared by western blot analysis in HEK293 cells. SGE, super gene expression; hTERT, human telomerase reverse transcriptase; SV40, Simian virus 40; REIC, reduced expression in immortalized cells; CMV, cytomegalovirus; Dkk-3, Dickkopf-3.

In the pShuttle plasmid vector system driven by the CMV promoter (Clontech Laboratories, Inc., Mountain View, CA, USA), we inserted the REIC/Dkk-3 gene as the gene of interest and constructed the pShuttle-REIC plasmid. The full-length cDNA of the human REIC/Dkk3 gene was amplified by PCR with primers containing *Xba*I and *Kpn*I restriction sites. The PCR product was cloned into the pShuttle vector to generate pShuttle-REIC. To construct the pShuttle-SGE-REIC plasmid, we inserted the sequences of the triple translational enhancers of hTERT, SV40 and CMV downstream of the BGH polyA sequence (Fig. 1A) of the pShuttle-REIC vector. The tandem sequences of the hTERT enhancer [189 bp: accession no. DQ264729 (1618–1806)], SV40 enhancer [319 bp: accession no. AY864928 (2156–2474)] and CMV enhancer [479 bp: accession no. AJ318513 (159–637)] were artificially synthesized and cloned into pShuttle-REIC at the restriction site between *Kpn*I and *Eco*RI.

### Construction and production of adenoviral vectors

In order to generate CMV promoter-driven Ad-CMV-REIC and Ad-SGE-REIC vectors, pShuttle-REIC and pShuttle-SGE-REIC plasmids were digested with the restriction enzymes I-*Ceu*I and PI-*Sce*I and inserted into the Adeno-X Viral DNA (Clontech Laboratories, Inc.). The recombinant adenoviral DNA with the full-length human REIC/Dkk-3 gene was linearized by *Pac*I digestion and transfected into HEK293 cells. Seven to ten days after transfection, the HEK293 cells were harvested, and a viral solution was obtained by three freeze/thaw cycles. The recovered virus solution was used to propagate sufficient viruses in HEK293 cells for further studies in other cell lines. All virus particles were purified by CsCl density gradient ultracentrifugation and stored at −80°C. For Ad-CAG-REIC under the control of the CAG promoter (CMV early enhancer/chicken β-actin promoter), the full-length human REIC/Dkk-3 gene was inserted into the cosmid vector, pAxCAwt, and then transferred into an adenoviral vector using the COS-TPC method (Takara Bio, Inc., Shiga, Japan) (16,17). An adenoviral vector carrying the LacZ gene with a CAG promoter (Ad-LacZ) was used as the control vector. These adenoviral vectors were generated using replication-defective adenoviruses of serotype 5.

### Western blot analysis

The cells (5.0×10^5^ cells) were plated in flat-bottom 6-well plates in complete culture medium, and after a 24-h incubation, transient transfections with the plasmid or adenoviral vectors were performed. During the plasmid transfection, HEK293 cells were transfected with the FuGENE HD reagent (Promega, Madison, WI, USA) and the cells were sampled 24 h after transfection. During the transfection with adenoviral vectors, the cells were treated with Ad-LacZ and Ad-REIC at the indicated multiplicity of infection (MOI) in 0.3 ml of complete medium for 1 h. Then, 1.7 ml of fresh medium was added and the cells were incubated for another 24 h. After the incubation, the attached cells were sampled, and a western blot analysis was performed as previously described (18,21). A mouse monoclonal anti-human REIC/Dkk-3 antibody raised in our laboratory (1:1,000) was used as the primary antibody.

### Apoptosis assay

The cells (5.0×10^5^ cells) were seeded in 6-well plates and incubated in culture medium for 24 h. The cells were then treated with Ad-LacZ and Ad-REIC at the indicated MOI, as described above. After 48 or 72 h of incubation, the apoptotic cells were visualized by Hoechst 33342 staining, and the apoptotic rate was analyzed as previously described (18,23).

### Subcutaneous tumor models and treatments

Mouse RENCA renal cell carcinoma cells [2×10^5^ cells/100 μl phosphate-buffered saline (PBS)] and human PC3 prostate cancer cells (2×10^6^ cells/100 μl PBS) were subcutaneously injected into the right thigh of female BALB/C mice and male athymic nude mice, respectively. The tumors were allowed to reach ~8 mm in diameter, and the mice were then randomly assigned to treatment groups of four or five animals each. In the tumor model with RENCA cells, there were five groups treated with a single intratumoral injection of: i) PBS (100 μl) or ii) Ad-LacZ, iii) Ad-CMV-REIC, iv) Ad-CAG-REIC or v) Ad-SGE-REIC adenoviral vectors at a dose of 5×10^9^ viral particles/100 μl PBS/injection. In the PC3 tumor model, there were four groups treated with a single injection or three intratumoral injections of: i) PBS (100 μl) single injection or ii) Ad-CAG-REIC single injection, iii) Ad-SGE-REIC single injection or iv) three injections of Ad-SGE-REIC on days 0, 2 and 4 at a dose of 1×10^10^ viral particles/100 μl PBS/injection. The tumor volume was calculated as previously described (18,19).

### Statistical analysis

The data are shown as the means ± standard error. An analysis of variance (ANOVA) was performed to determine the statistical significance of differences in the apoptosis rates. Unpaired Student’s t-tests were performed for the statistical analyses of the tumor volumes. Differences were considered to be statistically significant at P<0.05.

## Results

### The SGE system significantly enhances the REIC/Dkk-3 gene expression by the plasmid and adenoviral vectors

**Figure 2:**
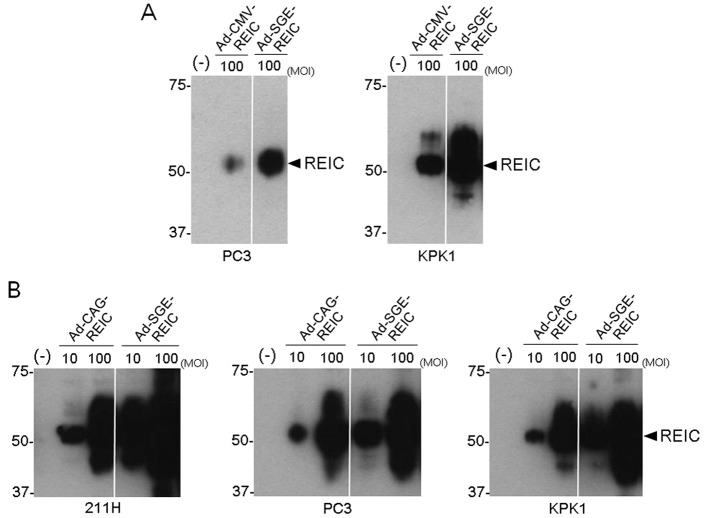
(A) The REIC/Dkk-3 expression levels after transfection at 100 MOI with CMV promoter-driven adenoviral vectors (Ad-CMV-REIC and Ad-SGE-REIC) were compared by western blot analysis in PC3 and KPK1 human cancer cells. (B) The REIC/Dkk-3 expression levels after transfection at 10 MOI and 100 MOI with Ad-CAG-REIC and Ad-SGE-REIC were compared by western blot analysis in 211H, PC3 and KPK1 human cancer cells. SGE, super gene expression; REIC, reduced expression in immortalized cells; CMV, cytomegalovirus; Dkk-3, Dickkopf-3; MOI, multiplicity of infection.

In order to demonstrate the enhanced gene expression induced by the SGE system, we constructed a pShuttle plasmid vector with the SGE system encoding the REIC/Dkk-3 gene (pShuttle-SGE-REIC) and compared the expression with that of the pShuttle-REIC plasmid vector with the conventional gene expression system. The REIC/Dkk-3 expression level after transfection with the pShuttle-SGE-REIC plasmid was significantly upregulated in comparison to that of the pShuttle-REIC in a western blot analysis of HEK293 cells (Fig. 1B). To further verify the broad utility of the SGE system, we put the SGE cassette in adenoviral vectors and developed an adenoviral vector expressing the REIC/Dkk-3 gene based on the CMV promoter-driven SGE system (Ad-SGE-REIC). We compared the REIC/Dkk-3 expression levels between the CMV promoter-driven adenoviral vectors (Ad-CMV-REIC and Ad-SGE-REIC) and demonstrated significantly enhanced REIC/Dkk-3 gene expression by the SGE system in the human cancer cells (Fig. 2A). In addition, in the comparison between the Ad-CAG-REIC and Ad-SGE-REIC vectors by a western blot analysis in multiple human cancer cell lines, robust upregulation of REIC/Dkk-3 expression was observed in the Ad-SGE-REIC-transfected cells, particularly after the transfection at 10 MOI (Fig. 2B).

### Significantly enhanced apoptosis is induced in various human cancer cell lines by Ad-SGE-REIC treatment

**Figure 3:**
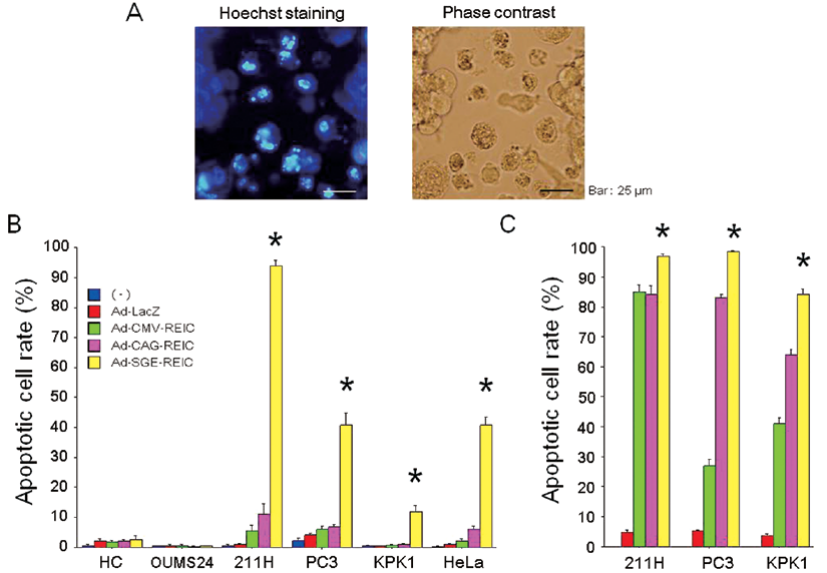
(A) The induction of apoptosis after Ad-REIC treatment is shown by Hoechst 33342 staining in 211H human malignant mesothelioma cells. Apoptotic cells can be clearly observed as bright cells under fluorescence microscopy (left panel). The appearance of the cells by phase contrast microscopy is also shown (right panel). (B) The apoptotic cell rate (%) was examined after the indicated treatments (no treatment, Ad-LacZ, Ad-CMV-REIC, Ad-CAG-REIC and Ad-SGE-REIC at 50 MOI for 48 h) in normal human cells (HC, hepatocytes; OUMS24, fibroblasts) and various human cancer cell lines (211H, PC3, KPK1 and HeLa). The apoptotic cell rate was determined in five different fields under microscopic observations. ^*^A significant difference was observed between the Ad-SGE-REIC group and the other treatment groups. (C) The apoptotic cell rate (%) was examined under different experimental conditions (Ad-LacZ, Ad-CMV-REIC, Ad-CAG-REIC or Ad-SGE-REIC at a 100 MOI for 72 h) in the human cancer cells (211H, PC3 and KPK1). ^*^A significant difference was observed between the Ad-SGE-REIC group and the other treatment groups. SGE, super gene expression; REIC, reduced expression in immortalized cells; CMV, cytomegalovirus; MOI, multiplicity of infection.

To examine the therapeutic utility of the SGE system and Ad-SGE-REIC vector in human cancer cells, we assessed the *in vitro* apoptotic effects of Ad-SGE-REIC in comparison to those of the Ad-CMV-REIC and Ad-CAG-REIC vectors using the conventional gene expression system. The Ad-SGE-REIC vector significantly enhanced the *in vitro* apoptosis induction in the various human cancer cells in comparison to the control vectors transfected at 50 MOI for 48 h (Fig. 3A and B). In the normal HC and fibroblasts (OUMS24), there was no significant apoptosis induction by any of the Ad-REIC vectors, as was previously reported for the Ad-CAG-REIC vector (16,18,20). Under the other experimental condition of 100 MOI for 72 h, significant apoptosis induction was confirmed following Ad-CMV-REIC and Ad-CAG-REIC treatment in comparison to the Ad-LacZ treatment (Fig. 3C). In addition, under these conditions, the induction of apoptosis was significantly enhanced in Ad-SGE-REIC-transfected cells compared to the cells transfected with conventional Ad-REIC vectors.

### Intratumoral Ad-SGE-REIC administration strongly inhibits the tumor growth in subcutaneous mouse tumor models

**Figure 4:**
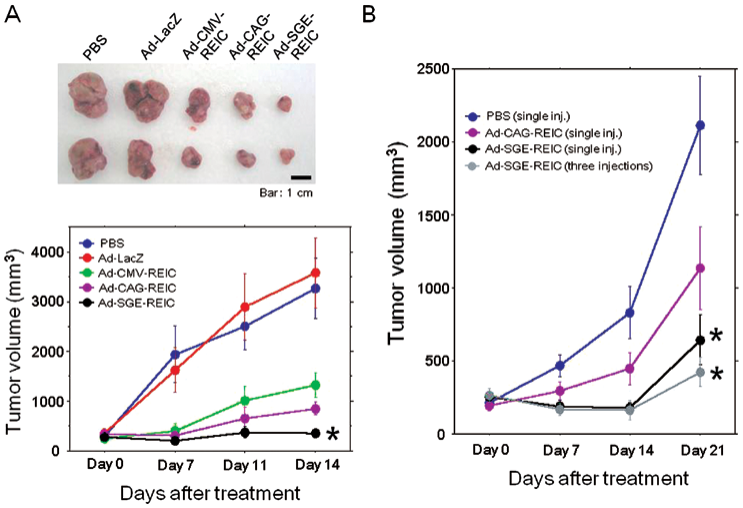
(A) The inhibitory effects of the Ad-REIC treatment on the subcutaneous tumor growth of RENCA mouse renal cell carcinoma cells in BALB/C mice. The representative appearance of the tumors at the end of the two-week observation period is shown (upper panel). The mean volume of tumors was calculated in four mice from each group, and the tumor growth curves are shown (lower panel). ^*^A significant difference was observed between the Ad-SGE-REIC group and the other treatment groups. (B) The inhibitory effects of the Ad-REIC treatment on the subcutaneous tumor growth of human PC3 prostate cancer cells in athymic nude mice. The mean volume of tumors was calculated in five mice from each group, and the tumor growth curves are shown. ^*^A significant difference was observed between the Ad-SGE-REIC groups (single injection and three injections) and the PBS treatment group. SGE, super gene expression; REIC, reduced expression in immortalized cells; PBS, phosphate-buffered saline.

To further evaluate the therapeutic utility of the SGE system and Ad-SGE-REIC vector, the inhibitory effects of Ad-REIC treatment on the subcutaneous tumor growth were analyzed in mouse tumor models. In both the mouse renal cell carcinoma (RENCA cells) (Fig. 4A) and human prostate cancer (PC3 cells) (Fig. 4B) models, strong suppression of tumor growth was observed in the Ad-SGE-REIC-treated groups in comparison to the other treatment groups. These results are consistent with the findings of the *in vitro* studies comparing the REIC/Dkk-3 gene expression and apoptosis induction by the three types of Ad-REIC treatment. Thus, the novel SGE system significantly enhances the therapeutic antitumor effects in mouse tumor models, and the Ad-SGE-REIC vector was superior to the conventional Ad-CMV-REIC and Ad-CAG-REIC vectors in terms of the efficacy of *in vivo* intratumoral gene therapy.

## Discussion

Gene therapy-based approaches often require strong levels of the gene expression and protein products. We have been developing methods to enhance the gene transcription driven by various promoters, and herein described a novel strategy to achieve higher levels of gene expression. We developed the SGE system, which enables the powerful gene expression, by putting the triple translational enhancer sequences of hTERT, SV40 and CMV downstream of the BGH polyA sequence. We herein demonstrated that the plasmid vector and adenoviral vector systems with a CMV promoter-driven SGE cassette can significantly enhance the expression of the CMV promoter, which is one of the strongest promoters reported (7,8).

The combination of multiple promoter/enhancer elements has been attempted in previous studies, with several showing improved gene transcription. The hTERT promoter/enhancer is well-characterized and has been frequently used for cancer-specific gene expression (2,12–14). In one of the studies using hTERT promoter/enhancer-driven plasmid vectors, the combination of the hTERT promoter with multiple hTERT enhancer regions significantly enhanced the gene expression in comparison to that of the parental plasmid (11). With regard to the SV40 enhancer, several studies demonstrated enhanced gene expression by the insertion of the SV40 enhancer downstream of the polyA sequences (7–10). The CMV enhancer is used in the CMV early enhancer/chicken β-actin promoter (CAG promoter), which is known to improve the gene expression in various cell types and tissues (3,4,7,8). The CMV enhancer is also used to stimulate the CMV promoter, EF-1α promoter and ubiquitin promoter, thereby further increasing the expression of the gene of interest (7,8,24). Therefore, each enhancer (hTERT, SV40 and CMV) can positively stimulate the promoters in a variety of vector systems and improve the gene expression levels in mammalian cells. The present study confirmed the utility of using the combination of the hTERT, SV40 and CMV enhancers in this order in the gene expression construct. The mechanism underlying the upregulated gene transcription by the hybrid promoter/enhancer system is not yet fully understood. It is conceivable that the transcriptional elements of the hTERT, SV40 and CMV triple enhancers interact individually or synergistically with the CMV promoter that drives the gene transcriptional complexes and upregulates the capacity of the cells for gene expression.

The REIC/Dkk-3 gene is a tumor suppressor and promising cancer therapeutic gene (16–23). To examine the possible use of REIC/Dkk-3 for targeted gene-based therapy, we developed a replication-deficient adenoviral vector encoding the human REIC/Dkk-3 gene (Ad-REIC) (16). The CAG promoter was selected to drive the REIC/Dkk-3 expression as it enables strong gene expression (3,4,7,8). The overexpression of REIC/Dkk-3 with the Ad-CAG-REIC reagent was previously found to induce apoptosis in a broad range of human cancer cell lines *in vitro* (16–18,23). On the other hand, the ability of Ad-CAG-REIC to induce apoptosis was lower in the non-malignant cells (16,18,20). These findings indicate that the Ad-CAG-REIC selectively induces apoptosis in a cancer cell-specific manner, and this was also demonstrated in the present study for the other Ad-REIC reagents (Ad-CMV-REIC and Ad-SGE-REIC). Since REIC/Dkk-3 expression is significantly downregulated in several human cancer cells, but is typically expressed in non-malignant or normal cells (15,16,18,21), the endogenous expression levels of the protein seem to be correlated with the sensitivity of the cells against the REIC/Dkk-3 overexpression by Ad-REIC.

In addition, the abundant REIC/Dkk-3 protein expression and secretion after intratumoral Ad-REIC administration could provide anticancer immunological effects via a mechanism involving autologous cancer vaccination (19,20). To further improve the therapeutic outcomes, we developed a new adenoviral vector expressing the REIC/Dkk-3 gene, based on the CMV promoter-SGE system (Ad-SGE-REIC), and compared the therapeutic utility of Ad-SGE-REIC with that of the conventional adenoviral vectors (Ad-CMV-REIC or Ad-CAG-REIC). In all the cancer cell lines tested in this study, the Ad-SGE-REIC was superior to conventional adenoviral systems in terms of the REIC/Dkk-3 protein expression and *in vitro* induction of apoptosis. With regard to the *in vivo* therapeutic effects, the antitumor effects of the Ad-SGE-REIC vector were also superior to those of the control adenoviral vectors, indicating the potential for promising therapeutic outcomes in future REIC/Dkk-3 gene therapy using Ad-SGE-REIC. Thus, the SGE system could be successfully applied to the adenovirus-mediated gene expression system, and might be useful as gene therapy for a wide range of human malignancies.

We herein demonstrated that the CMV promoter-driven SGE cassette is superior to the conventional systems with CMV and CAG promoters in terms of the vector-mediated gene expression. The novel hybrid promoter/enhancer system is a promising gene therapeutic cassette that can be used in combination with various vector systems, and has important practical implications for cancer gene therapy. We believe that this novel gene expression system will become an innovative tool in the fields of both gene expression and gene therapy.

## References

[1] Nasu Y, Saika T, Ebara S, Kusaka N, Kaku H, Abarzua F, Manabe D, Thompson TC, Kumon H (2007). Suicide gene therapy with adenoviral delivery of HSV-tK gene for patients with local recurrence of prostate cancer after hormonal therapy. Mol Ther.

[2] Fukazawa T, Matsuoka J, Yamatsuji T, Maeda Y, Durbin ML, Naomoto Y (2010). Adenovirus-mediated cancer gene therapy and virotherapy (Review). Int J Mol Med.

[3] Niwa H, Yamamura K, Miyazaki J (1991). Efficient selection for high-expression transfectants with a novel eukaryotic vector. Gene.

[4] Araki K, Araki M, Miyazaki J, Vassalli P (1995). Site-specific recombination of a transgene in fertilized eggs by transient expression of Cre recombinase. Proc Natl Acad Sci USA.

[5] Ebara S, Shimura S, Nasu Y, Kaku H, Kumon H, Yang G, Wang J, Timme TL, Aguilar-Cordova E, Thompson TC (2002). Gene therapy for prostate cancer: toxicological profile of four HSV-tk transducing adenoviral vectors regulated by different promoters. Prostate Cancer Prostatic Dis.

[6] Watanabe M, Nasu Y, Kashiwakura Y, Kusumi N, Tamayose K, Nagai A, Sasano T, Shimada T, Daida H, Kumon H (2005). Adeno-associated virus 2-mediated intratumoral prostate cancer gene therapy: long-term maspin expression efficiently suppresses tumor growth. Hum Gene Ther.

[7] Xu ZL, Mizuguchi H, Ishii-Watabe A, Uchida E, Mayumi T, Hayakawa T (2001). Optimization of transcriptional regulatory elements for constructing plasmid vectors. Gene.

[8] Xu ZL, Mizuguchi H, Ishii-Watabe A, Uchida E, Mayumi T, Hayakawa T (2002). Strength evaluation of transcriptional regulatory elements for transgene expression by adenovirus vector. J Control Release.

[9] Young JL, Benoit JN, Dean DA (2003). Effect of a DNA nuclear targeting sequence on gene transfer and expression of plasmids in the intact vasculature. Gene Ther.

[10] Song JS (2005). Enhanced expression of apoptin by the Myc-Max binding motif and SV40 enhancer for SCLC gene therapy. Biosci Biotechnol Biochem.

[11] Kim SJ, Lee HS, Shin JH, Kim CG, Jeong S, Park K, Choe H, Lee H (2006). Preferentially enhanced gene expression from a synthetic human telomerase reverse transcriptase promoter in human cancer cells. Oncol Rep.

[12] Kojima T, Hashimoto Y, Watanabe Y, Kagawa S, Uno F, Kuroda S, Tazawa H, Kyo S, Mizuguchi H, Urata Y, Tanaka N, Fujiwara T (2009). A simple biological imaging system for detecting viable human circulating tumor cells. J Clin Invest.

[13] Watanabe M, Ueki H, Ochiai K, Huang P, Kobayashi Y, Nasu Y, Sasaki K, Kaku H, Kashiwakura Y, Kumon H (2011). Advanced two-step transcriptional amplification as a novel method for cancer-specific gene expression and imaging. Oncol Rep.

[14] Ueki H, Watanabe M, Kaku H, Huang P, Li SA, Ochiai K, Hirata T, Noguchi H, Yamada H, Takei K, Nasu Y, Kashiwakura Y, Kumon H (2012). A novel gene expression system for detecting viable bladder cancer cells. Int J Oncol.

[15] Tsuji T, Miyazaki M, Sakaguchi M, Inoue Y, Namba M (2000). A REIC gene shows down-regulation in human immortalized cells and human tumor-derived cell lines. Biochem Biophys Res Commun.

[16] Abarzua F, Sakaguchi M, Takaishi M, Nasu Y, Kurose K, Ebara S, Miyazaki M, Namba M, Kumon H, Huh NH (2005). Adenovirus-mediated overexpression of REIC/Dkk-3 selectively induces apoptosis in human prostate cancer cells through activation of c-Jun-NH2-kinase. Cancer Res.

[17] Kashiwakura Y, Ochiai K, Watanabe M, Abarzua F, Sakaguchi M, Takaoka M, Tanimoto R, Nasu Y, Huh NH, Kumon H (2008). Down-regulation of inhibition of differentiation-1 via activation of activating transcription factor 3 and Smad regulates REIC/Dickkopf-3-induced apoptosis. Cancer Res.

[18] Kawasaki K, Watanabe M, Sakaguchi M, Ogasawara Y, Ochiai K, Nasu Y, Doihara H, Kashiwakura Y, Huh NH, Kumon H, Date H (2009). REIC/Dkk-3 overexpression downregulates P-glycoprotein in multidrug-resistant MCF7/ADR cells and induces apoptosis in breast cancer. Cancer Gene Ther.

[19] Watanabe M, Kashiwakura Y, Huang P, Ochiai K, Futami J, Li SA, Takaoka M, Nasu Y, Sakaguchi M, Huh NH, Kumon H (2009). Immunological aspects of REIC/Dkk-3 in monocyte differentiation and tumor regression. Int J Oncol.

[20] Sakaguchi M, Kataoka K, Abarzua F, Tanimoto R, Watanabe M, Murata H, Than SS, Kurose K, Kashiwakura Y, Ochiai K, Nasu Y, Kumon H, Huh NH (2009). Overexpression of REIC/Dkk-3 in normal fibroblasts suppresses tumor growth via induction of interleukin-7. J Biol Chem.

[21] Zhang K, Watanabe M, Kashiwakura Y, Li SA, Edamura K, Huang P, Yamaguchi K, Nasu Y, Kobayashi Y, Sakaguchi M, Ochiai K, Yamada H, Takei K, Ueki H, Huh NH, Li M, Kaku H, Na Y, Kumon H (2010). Expression pattern of REIC/Dkk-3 in various cell types and the implications of the soluble form in prostatic acinar development. Int J Oncol.

[22] Ochiai K, Watanabe M, Ueki H, Huang P, Fujii Y, Nasu Y, Noguchi H, Hirata T, Sakaguchi M, Huh NH, Kashiwakura Y, Kaku H, Kumon H (2011). Tumor suppressor REIC/Dkk-3 interacts with the dynein light chain, Tctex-1. Biochem Biophys Res Commun.

[23] Hirata T, Watanabe M, Kaku H, Kobayashi Y, Yamada H, Sakaguchi M, Takei K, Huh NH, Nasu Y, Kumon H (2012). REIC/Dkk-3-encoding adenoviral vector as a potentially effective therapeutic agent for bladder cancer. Int J Oncol.

[24] Wang CY, Guo HY, Lim TM, Ng YK, Neo HP, Hwang PY, Yee WC, Wang S (2005). Improved neuronal transgene expression from an AAV-2 vector with a hybrid CMV enhancer/PDGF-beta promoter. J Gene Med.

